# Acute acalculous cholecystitis as the initial manifestation of systemic lupus erythematous: A case report

**DOI:** 10.1097/MD.0000000000026238

**Published:** 2021-06-04

**Authors:** Jeonghun Lee, Young Joo Lee, Youngsun Kim

**Affiliations:** a Department of Internal Medicine, Ye Hospital, Anyang; b Department of Obstetrics and Gynecology, Kyung Hee University Medical Center, Kyung Hee University College of Medicine, Seoul, Korea.

**Keywords:** acute acalculous cholecystitis, corticosteroid, initial manifestation, systemic lupus erythematous

## Abstract

**Rationale:**

Acute acalculous cholecystitis (AAC) is an extremely rare manifestation of systemic lupus erythematous (SLE). In previous reports, most of the patients were already diagnosed cases of SLE upon confirmation of AAC.

**Patient concerns:**

A 24-year-old female who initially presented with fever and acute right upper quadrant abdominal pain. She had no medical history.

**Diagnoses:**

Abdominal ultrasonography and computed tomography (CT) showed gallbladder thickening with pericholecystic edema without gallstones or sludge, demonstrating acalculous cholecystitis. She revealed discoid rash on the both shin. Laboratory tests revealed pancytopenia. The titer of antinuclear antibody (ANA) was 1:1280. Anti-dsDNA antibody, anti-phospholipid antibody, anti-Sm antibody test, and proteinuria in 24 hours were positive. Both C3 and C4 were low. Echocardiography and chest CT showed pericardial effusion and pleural effusion. Using the 2019 European League Against Rheumatism/American College of Rheumatology (EULAR/ACR) classification criteria, the score was 31. We thought AAC of this case that was one of the initial manifestations of SLE.

**Interventions:**

The patient was treated with high-dose prednisolone (1 mg/kg) and hydroxychloroquine 400 mg.

**Outcomes:**

After 4 days of administration of high-dose corticosteroid therapy, symptoms rapidly improved. After 35 days of the treatment, her symptoms and disease activity of SLE were markedly improved.

**Lessons:**

Although AAC being the initial manifestation of SLE is very rare, prompt diagnosis and management with corticosteroids precluded surgical intervention. Physicians need to be cognizant of AAC as a disease flare and as a rare initial manifestation of SLE.

## 1 Introduction

Most acute acalculous cholecystitis (AAC) occurring in critically ill patients likely exhibit a worse prognosis than acute calculous cholecystitis in immunocompetent individuals. Cholecystectomy is often considered the definitive treatment because of its high morbidity and mortality.^[1]^ Most cases of AAC in systemic lupus erythematosus (SLE) developed after the SLE diagnosis. AAC is a very rare initial manifestation in SLE, and it is difficult to recognize that AAC is associated with SLE. Recently, high dose corticosteroid therapy has been a successful treatment regimen in patients with AAC in SLE due to the improvement of symptoms and absence of infection-related complications.^[2]^ However, there is no definitive consensus regarding the treatment of AAC in SLE. We report a case of a 24-year-old female with AAC as an initial manifestation of SLE who demonstrated improvement of symptoms and disease activity after administration of high-dose corticosteroid therapy.

## 2 Case report

This study was approved by the Ethics Committee and Institutional Review Board of the Kyung Hee University Medical Center, Seoul, Korea. The patient provided informed consent for publication of this case.

A 24-year-old female was admitted to our hospital with fever and acute right upper quadrant abdominal pain. The patient had no family history of hepatobiliary disease.

**Figure 1:**
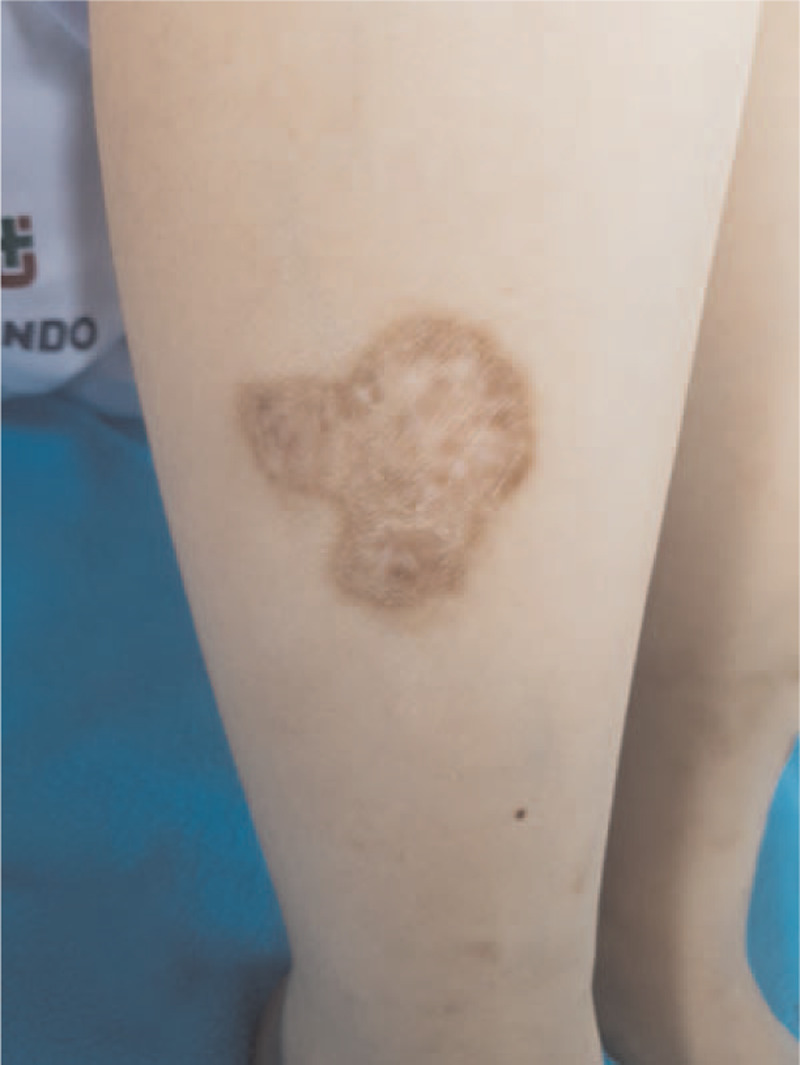
The discoid rash revealed irregular disc-shaped, dark erythematous plaques containing hyperkeratosis on shin.

On admission, blood pressure was 130/90 mmHg, body temperature was 38.8°C, pulse rate was 72/min, and respiratory rate was 20/min. A discoid rash was noted on both her shins (Fig. 1).

On physical examination, tenderness was appreciated in the right upper quadrant of her abdomen and Murphy's sign was positive. Laboratory tests revealed pancytopenia (white blood cells 3860/μL, hemoglobin 5.2 g/dL, and platelet 107,000/μL) with normal hepatobiliary enzymes (aspartate aminotransferase, 35 IU/L; alanine aminotransferase, 11 IU/L; total bilirubin, 0.32 mg/dL; gamma-glutamyl transferase, 33 U/L; alkaline phosphatase, 96 U/L; and albumin, 3.0 g/dL). Serum creatinine and procalcitonin levels were normal at 0.81 mg/dL and 0.24 ng/mL, respectively. CRP and ESR were elevated at 65.33 mg/dL and >150 mm/h, respectively. NT-proBNP level was elevated at 4920 pg/mL. Low complement levels (C3 54 mg/dL, C4 5 mg/dL) and high antinuclear antibody titer (ANA; 1:1280, homogenous pattern) with positive anti-dsDNA antibody (>98.00 IU/mL), anti-phospholipid antibody, and anti-U1-RNP antibody (10.9 IU/mL) were revealed. Anti-SSA antibody was positive, but the anti-SSB antibody, anti-Sm antibody, MPO-ANCA and PR3-ANCA, and direct/indirect Coombs were negative. Urine analysis was positive for hematuria and 3+ proteinuria (819 mg in 24 hours).

**Figure 2:**
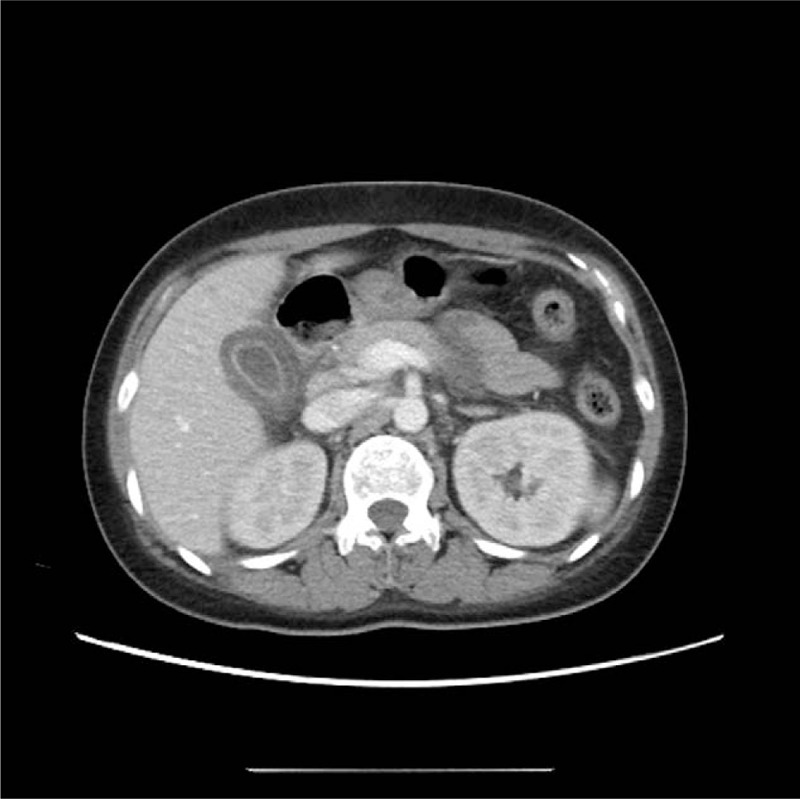
Abdominal computed tomography findings. Edematous gall bladder wall thickening without stones.

**Figure 3:**
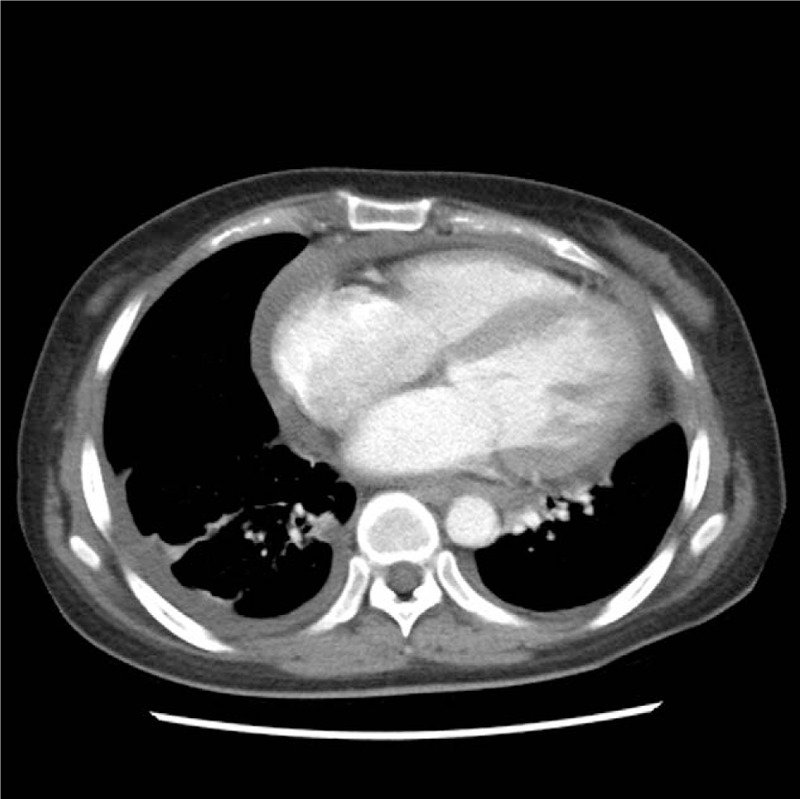
Chest computed tomography findings. Cardiomegaly with pericardial effusion, bilateral pleural effusions, probably loculate, without discernible pleural thickening.

Abdominal ultrasonography and CT showed gallbladder thickening with pericholecystic edema without gallstones or sludge, demonstrating acalculous cholecystitis (Fig. 2). Transthoracic echocardiography and chest CT showed pericardial effusion and pleural effusion, indicating pericarditis and pleuritis as complications (Fig. 3).

Based on the clinical criteria which include a discoid rash, hair fragility with broken hairs, pleural effusion, pericardial effusion, proteinuria, leukopenia, and thrombocytopenia, and immunologic markers such as positive ANA, anti-dsDNA antibody, antiphospholipid antibody, and low complement levels, she was diagnosed with active SLE, fulfilling 10 points from the Systemic Lupus International Collaborating Clinics 2012 criteria and 31 points from the European League Against Rheumatism/American College of Rheumatology (EULAR/ACR) 2019 classification criteria. We surmised that a significant infection was not the precipitating factor because her condition did not deteriorate, and there was no evident increase in procalcitonin values, and the blood culture was rendered negative.

**Figure 4:**
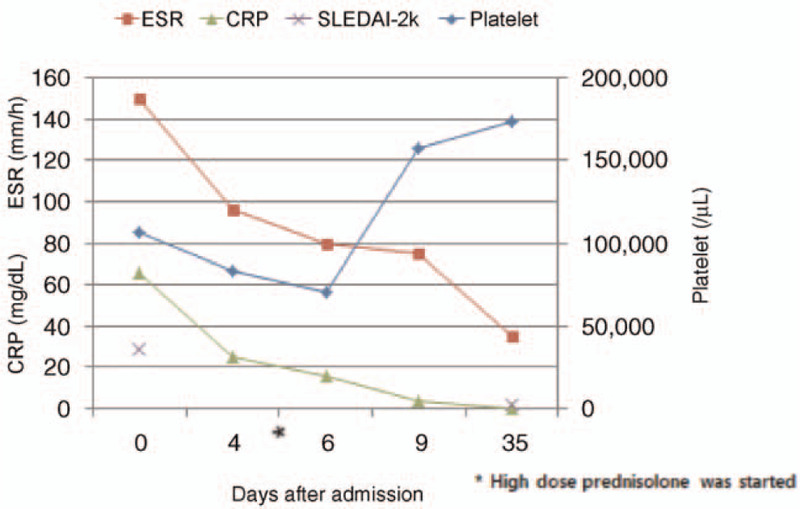
Repeated laboratory tests showed improvement after corticosteroid treatment.

Since previous reports of AAC in SLE were associated with an active disease flare, we considered AAC in this case as one of the manifestations of SLE (SLEDAI-2K 29 points). Therefore, we administered high-dose prednisolone (1 mg/kg) and hydroxychloroquine 400 mg for the treatment of AAC in SLE. After 4 days of the treatment, the symptoms and laboratory tests were improved. Follow-up CT and ultrasonography were preformed 1 month after discharge and revealed no abnormal findings. In addition, disease activity markedly improved (SLEDAI-2K 2 points) (Fig. 4). The patient has been treated with hydroxychloroquine and corticosteroid tapering.

## 3 Discussion

SLE is an autoimmune disease characterized by systemic clinical manifestations. Various manifestations of vasculitis occur in association with SLE. The diagnosis of SLE is mostly proposed by the Systemic Lupus International Collaborating Clinics 2012 and European League Against Rheumatism/American College of Rheumatology (EULAR/ACR) 2019 criteria.^[3,4]^ The EULAR/ACR 2019 criteria have superior specificity in early SLE.^[5]^ In our case, the titer of ANA was 1:1280. She had a fever (point: 2) and thrombocytopenia with anemia (point: 4). Moreover, a discoid rash was noted on both shins (point: 4). Echocardiography and chest CT showed pericardial effusion and pleural effusion (point: 5). Proteinuria within 24 hours, anti-phospholipid antibody, and anti-Sm antibody tests were positive (respectively, point: 4; 2; 6). Both C3 and C4 levels were low (point 4). Using the 2019 EULAR/ACR classification criteria, the score was 31.

Gastrointestinal manifestations of SLE are rather common, but gallbladder involvement is rare.^[6]^ AAC had an incidence of less than 0.05% as the initial manifestation in patients with SLE.^[7]^ The diagnosis of AAC was based on the clinical manifestations, ultrasonography, CT, and hepatobiliary iminodiacetic acid scans.^[1,8]^ In our patient, abdominal ultrasonography and CT showed an inflamed gallbladder with thickened walls and pericholecystic fluid without gallstones.

Although the pathogenesis of SLE-associated AAC is still unknown, vasculitis, thrombosis, and mesenteric inflammatory veno-occlusive disease (MIVOD), and serositis could be the pathogenesis.^[9–12]^ Vasculitis is considered the most common pathogenesis of SLE-associated AAC. Arteritis and venulitis in the gallbladder may be caused by the aggravation of SLE. The deposition of immune complexes in the blood vessel walls may lead to vasculitis of the target organ.^[13,14]^ Thrombosis may be observed in SLE patients with antiphospholipid antibodies. This is characterized histologically by multiple thrombi and no evidence of vasculitis.^[11]^ MIVOD is another rare cause of AAC in patients with SLE. This type of vasculitis completely involves the mesenteric veins or their branches and sparing arteries. The main inflammatory cells are lymphocytes with occasional granulomatous inflammation, but the etiology of MIVOD is unclear.^[7,10]^ Although the gallbladder lacks serosa on the surface attached to the liver, serositis may rarely cause cholecystitis.^[12,14]^ In our case, intestinal ischemia was not detected on abdominal CT. Although the antiphospholipid antibody test was positive and there were no pathologic findings, vasculitis was considered as the main pathogenesis for this case because of the rapid improvement with corticosteroids.

There are only a few studies on initial manifestation as AAC in SLE. Usually, AAC is a severe disease flare in patients who are identified to have SLE. AAC is a severe disease flare in patients who are identified to have SLE. We searched PubMed on cases related to SLE-associated AAC, published after the year 2000, using the phrase “systemic lupus erythematosus and acute acalculous cholecystitis.” We found only 7 case reports and 10 patients (Table 1). All patients were women, and their average age was 34.9 years. The mean SLE Disease Activity Index 2000 was 13.7. Almost all patients were treated with high-dose corticosteroids, except 2 patients who required cholecystectomy. All patients were successfully treated.

**Table 1:** Previous reports of acute acalculous cholecystitis as the initial manifestation in an undiagnosed systemic lupus erythematous.

Case/Reference	Age (years)	Sex	SLEDAI-2k	Treatment	Outcome
Yang H, et al ^∗∗^	29	F	9	CS/CTX	Remission
	26	F	4	CS/HCQ	Remission
	43	F	15	CS/CTX	Remission
	32	F	20	CS/CTX/IVIG	Remission
Mendonca JA, et al ^∗∗^	12	F	NA	CS/AZA	Remission
Manuel V, et al ^∗∗^	20	F	9	CS	Remission
Kudo N, et al ^∗∗^	69	F	27	CS/HCQ/CSA/AZA	Remission
Choi YJ, et al ^∗∗^	70	F	20	CS/CTX	Remission
Obreja EI, et al ^∗∗^	22	F	9	Cholecystectomy/CS/HCQ	Remission
Mohapatra S, et al ^∗∗^	26	F	10	Cholecystectomy/CS/MMF	Remission
Current case	24	F	29	CS/HCQ	Remission

The treatment of SLE associated with AAC has been controversial. AAC has high morbidity and mortality, and is difficult to diagnose based on clinical and laboratory findings alone. Because of the high morbidity associated with AAC, cholecystectomy has been considered in the past.^[15]^ However, corticosteroid therapy has been successfully used as the initial treatment.^[2,7,16–18]^ Hydroxychloroquine allows the reduction of the dosage of corticosteroids and may reduce the risk of flares, organ damage. Most of patients were in remission after treatment without recurrence. However, there was also a case requiring subsequent cholecystectomy among previously reported cases treated with corticosteroids.^[18]^ The patient's general condition and risk factors are important in the medical or surgical treatment decisions. In our case, because the patient was stable and had no risk factors for AAC complications, we considered high-dose corticosteroid with hydroxychloroquine as the initial treatment for SLE-associated AAC. High-dose corticosteroids play a role in immune suppression and anti-inflammation for severe disease flare or inflammation of SLE.

This was a case of a patient who presented with AAC as the initial manifestation of SLE, who was treated successfully with high-dose corticosteroids and hydroxychloroquine. Although AAC is extremely rare as the initial manifestation of SLE, it is important to consider AAC as an initial manifestation or severe flare of SLE. As the first treatment for SLE associated AAC, high-dose corticosteroids should be used depending on the patient's general condition and risk factors.

## Author contributions

**Conceptualization:** Jeonghun Lee, Young Joo Lee.

**Investigation:** Youngsun Kim.

**Project administration:** Jeonghun Lee, Young Joo Lee.

**Resources:** Jeonghun Lee, Young Joo Lee.

**Software:** Jeonghun Lee, Young Joo Lee.

**Supervision:** Youngsun Kim.

**Writing – original draft:** Youngsun Kim, Jeonghun Lee, Young Joo Lee.

**Writing – review & editing:** Youngsun Kim.
